# The impact of hearing loss on annual incident age-associated dementia cases and quality of life in the United States

**DOI:** 10.1093/gerona/glaf295

**Published:** 2026-02-03

**Authors:** Ethan D Borre, Julie N Deleger, Lauren K Dillard, Juliessa M Pavon, Sachin J Shah, Judy R Dubno, Sherri L Smith, Kenneth A Freedberg, Howard W Francis, Christine S Ritchie, Gillian D Sanders Schmidler, Emily P Hyle

**Affiliations:** Medical Practice Evaluation Center, Massachusetts General Hospital, Boston, Massachusetts, United States; Duke-Margolis Institute for Health Policy, Duke University, Durham, North Carolina, United States; Duke-Margolis Institute for Health Policy, Duke University, Durham, North Carolina, United States; Department of Otolaryngology-Head and Neck Surgery, Medical University of South Carolina, Charleston, South Carolina, United States; Division of Geriatrics, Department of Medicine, Duke University School of Medicine, Durham, North Carolina, United States; Department of Head and Neck Surgery & Communication Sciences, Duke University School of Medicine, Durham, North Carolina, United States; Center for Aging and Serious Illness, Massachusetts General Hospital, Boston, Massachusetts, United States; Harvard Medical School, Harvard University, Boston, Massachusetts, United States; Department of Otolaryngology-Head and Neck Surgery, Medical University of South Carolina, Charleston, South Carolina, United States; Department of Head and Neck Surgery & Communication Sciences, Duke University School of Medicine, Durham, North Carolina, United States; Medical Practice Evaluation Center, Massachusetts General Hospital, Boston, Massachusetts, United States; Harvard Medical School, Harvard University, Boston, Massachusetts, United States; Department of Head and Neck Surgery & Communication Sciences, Duke University School of Medicine, Durham, North Carolina, United States; Center for Aging and Serious Illness, Massachusetts General Hospital, Boston, Massachusetts, United States; Harvard Medical School, Harvard University, Boston, Massachusetts, United States; Duke-Margolis Institute for Health Policy, Duke University, Durham, North Carolina, United States; Department of Population Health Sciences, Duke University School of Medicine, Durham, North Carolina, United States; Medical Practice Evaluation Center, Massachusetts General Hospital, Boston, Massachusetts, United States; Harvard Medical School, Harvard University, Boston, Massachusetts, United States

**Keywords:** Hearing loss, Dementia, Decision modeling

## Abstract

**Background:**

One-third of persons age 60 y+ have hearing loss, and hearing loss is a leading preventable risk factor for dementia. We estimated the number of age-associated dementia cases attributable to hearing loss in 2022.

**Methods:**

We used DeciBHAL, a validated microsimulation of hearing loss that includes age- and sex-specific annual probabilities of incident hearing loss (0.1%-10.4%) and dementia (0.3%-7.1%). Utility decrements are incorporated yearly, based on hearing loss (−0.13 to −0.31) and dementia severity (−0.04 to −0.42), to calculate quality-adjusted life-years (QALYs). We estimated dementia incidence for persons with and without hearing loss by removing the estimated proportion attributable to hearing loss (adjusted incidence risk ratio, 2.0 [range: 1.5-2.5]). We projected two cohorts: the general US population and a hypothetical US population without hearing loss (counterfactual). We applied model-projected dementia incidence and utility among both cohorts to the 74 190 000 US adults >60 y and without dementia in 2022.

**Results:**

Model-projected incident cases of dementia are 412 000/year (males) and 523 000/year (females). In the simulation without hearing loss, dementia cases/year fall to 339 000 for males and 455 000 for females projecting that 141 000 new dementia cases in 2022 would be attributable to hearing loss. In probabilistic sensitivity analysis, 95% of simulations projected the proportion of dementia cases attributable to hearing loss were 11.5%-23.6% for males and 6.7%-18.7% for females. Hearing loss and associated dementia reduced life-time QALYs by 1.38 for females and 1.69 for males.

**Conclusion:**

Model-projected estimates support that hearing loss prevention could substantially reduce new dementia cases and should be a priority.

## Introduction

Hearing loss and dementia are prevalent and morbid conditions that affect older adults. Over two-thirds of individuals aged over 70 years have any level of hearing loss, and 14% have dementia.^1–3^ Hearing loss is estimated to account for approximately 7% of the global burden of dementia, and recent evidence demonstrates that hearing loss is among the most preventable risk factors for dementia.^4^ In the United States, population-level data suggest that moderate to severe hearing loss is associated with an adjusted prevalence ratio of age-associated dementia (dementia) of nearly 2-fold.^5^ The mechanism(s) underlying the association of dementia and hearing loss is not currently known, but some hypotheses suggest that it could be related to maladaptive neuroplasticity in response to altered neural input or the social effects of hearing loss such as isolation and loneliness.^6^ As there are currently no treatments or interventions that cure or reverse dementia, ­prevention or delay of dementia onset is critical.

Both hearing loss and dementia significantly affect daily functioning and health-related quality of life. Hearing loss is the fourth leading contributor to disability-adjusted life-years globally, and untreated hearing loss negatively affects daily communication, increases loneliness and isolation, and increases risks of falls and hospitalization.^7^ Dementia progression is characterized by cognitive, behavioral, and functional decline, such as memory loss, communication problems, reasoning difficulties, personality changes, and the deterioration in the ability to carry out activities of daily living.^8^ This decline can severely affect daily functioning and health-related quality of life, as individuals may struggle with basic tasks such as dressing and eating, managing medications, and can also experience increased isolation due to communication challenges. Prior research has demonstrated that dementia and hearing loss alone have considerable impacts on quality of life, but few studies examine the combined effects of hearing loss and associated dementia on lifetime quality of life.^9^^,^^10^

Previous studies using population attributable fraction (PAF) methods have estimated that approximately 17% of dementia in the United States is attributable to moderate or greater hearing loss.^9^^,^^10^ While estimates exist on the risk of dementia onset due to hearing loss, the yearly number of incident dementia cases attributed directly or indirectly to hearing loss in the United States remains unknown. Further, little is known about the combined quality of life effects of hearing loss and dementia across the lifespan. Our simulation modeling approach expands on previous PAF studies to take into account the incidence of hearing loss and dementia, the relative risk of dementia given hearing loss, the impact of dementia and hearing loss on quality of life, and increases in mortality associated with dementia. Our objective was to estimate the sex-stratified number of dementia cases attributable to hearing loss and the impact of hearing loss and dementia on quality-adjusted life expectancy in the United States. As a secondary objective, we sought to estimate the healthcare costs associated with hearing loss and related dementia given the high costs of hearing and dementia care in the United States.^11^^,^^12^ Such information can help to establish and prioritize clinical and public health dementia prevention strategies.

## Methods

### Analytic overview

We used a previously validated model of hearing loss natural history, detection, diagnosis, and treatment (Decision model of the Burden of Hearing loss Across the Lifespan: DeciBHAL-US) to simulate the impact of hearing loss on the incidence of dementia.^13^ Based on epidemiologic patterns of adult hearing loss and dementia in the United States, we simulated 40-year-old males and females without hearing loss throughout their remaining lifetime. In the model, simulated persons experience yearly probabilities of incident hearing loss, subsequent hearing loss diagnosis, and hearing aid and cochlear implant uptake and discontinuation.^14^ Simulated persons also experience yearly probabilities of incident dementia progressing from mild to moderate and moderate to severe disease stages. The risk of incident dementia increases if the simulated person has better-ear hearing loss pure-tone average (PTA at frequencies 500, 1000, 2000, and 4000 Hz) of HL ≥ 40 dB HL.^1^^,^^5^^,^^13^^,^^15^ Quality-of-life utilities, based on hearing loss and dementia severity, were summed over the lifetime. We simulated four cohorts to ascertain the dementia cases and quality-of-life effects due to hearing loss alone: males with no incident hearing loss, males with age-stratified hearing loss incidence, females with no incident hearing loss, and females with age-stratified hearing loss incidence. All costs are reported in undiscounted 2022 US dollars from the healthcare sector perspective.

#### Model overview

**Figure 1.**
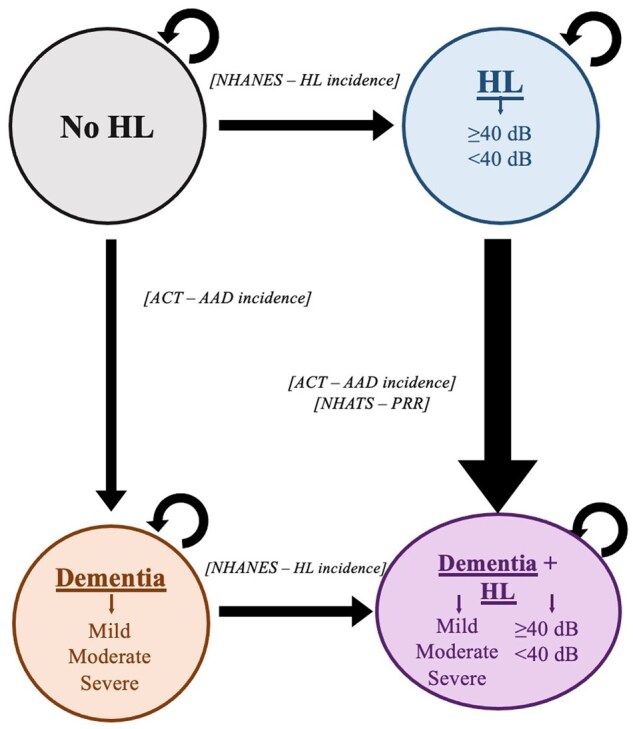
Health state diagram for age-associated dementia (dementia) and hearing loss (HL). This health state transition schematic shows the incorporation of dementia into the Markov microsimulation model, DeciBHAL-US, where each circle represents a distinct health state, and arrows represent transition probabilities. This figure specifically illustrates the health states related to no HL, dementia, HL, and dementia and HL. Simulated persons experience yearly probabilities of acquiring HL (HL incidence derived from the National Health and Nutrition Examination Survey, NHANES) and/or dementia (dementia incidence from the Adult Changes in Thought Cohort, ACT). The model incorporates an increased risk for dementia among persons with hearing losses greater than or equal to 40 dB hearing level, derived from the National Health and Aging Trends Study (NHATS). Simulated persons with dementia progress through mild, moderate, and severe disease stages in the model. Similarly, simulated persons with hearing loss experience annual decrements in their hearing level. For simplicity, non-sensorineural hearing loss, treated hearing loss, and death health states are not shown in the schematic. Abbreviations: ACT, Adult Changes in Thought; dB HL, decibels in hearing level; HA, hearing aids; HL, hearing loss; dementia, age-associated dementia; NHANES, National Health and Nutrition Examination Survey; NHATS, National Health and Aging Trends Study; PRR, prevalence risk ratio.

We expanded the hearing simulation model DeciBHAL to include dementia (Figure 1).^13^ The model simulates individuals on a yearly basis, considering factors such as age and sex to project health events. In DeciBHAL, simulated individuals face yearly probabilities of acquiring sensorineural, conductive hearing loss, or both. Subsequent hearing aid or cochlear implant adoption depends on age and hearing loss severity. Simulated persons also face a yearly probability of hearing loss treatment discontinuation, which decreases with prolonged amplification use. People with sensorineural hearing loss experience an annual age-related decline in hearing levels.^14^ Costs and quality-adjusted life years (QALYs) accrue based on hearing loss severity and treatment status. Throughout their lifetime, simulated persons acquire dementia at annual incidences determined by their age, sex, and hearing loss status (if greater than or equal to PTA 40 dB HL). Simulated persons also transition through different stages of dementia, from mild to moderate and moderate to severe. Each of these dementia stages triggers an increasing mortality multiplier applied to the simulated person’s age-/sex-stratified mortality. The dementia module in DeciBHAL was validated internally and externally to Adult Changes in Thought cohort data (Supplementary 1).^1^^,^^15^

#### Estimating hearing loss-attributable dementia

We estimated the number of dementia cases attributable to hearing loss by simulating cohorts of males and females with hearing loss incidence set to current United States estimates (HL+) and then a counterfactual scenario with no hearing loss incidence (HL−). The model incorporates the hearing loss-specific incidence risk ratio for incident dementia. The difference in incidence of dementia cases between the HL+ and HL− scenarios is the number of cases attributed directly to hearing loss. We then applied these model-projected differences to the number of people alive and at risk for dementia in each age/sex stratum.^3^^,^^14^^,^^16^

### Model input data

#### Natural history and treatment of hearing loss

Annual probabilities of bilateral sensorineural hearing loss, specific to age and sex, were derived from NHANES years 2001-2010 (0.8%-10.4% for males and 0.1%-9.2% for females; Table 1).^1^^,^^14^^,^^17^^,^^18^ Simulated individuals face a yearly increase in mean PTA hearing threshold, of 1.1-1.4 dB per year.^19^ We defined any hearing loss as a PTA hearing threshold ≥25 dB HL in the better ear. Hearing loss treatment includes hearing aids and cochlear implants. The age- and severity-specific annual probabilities of hearing aid uptake after acquiring hearing loss ranges from 0.5%-8.1%, increasing with age and hearing loss severity, and the probability of cochlear implant uptake for persons with moderate or severe hearing loss is 1% per year.^20–22^ After uptake of hearing aids, simulated persons experience an annual probability of hearing aid discontinuation (4%-13%) that decreases with time since acquisition.^23^^,^^24^ Similarly, persons with cochlear implants experience a 1% annual probability of discontinuation.^37^

**Table 1.** Selected model inputs for an analysis of the incident age-associated dementia cases attributable to hearing loss.

Input parameter		Base case value			Reference
**Hearing loss clinical input parameters**					
**Bilateral SNHL annual incidence (%)**					
	Male			Female	
** Ages 40-45 years**	0.8			0.1	^1^ ^,^ ^13^ ^,^ ^17^ ^,^ ^18^
** Ages 46-55 years**	1.2			0.4	
** Ages 56-65 years**	2.3			1.3	
** Ages 66-75 years**	5.4			3.8	
** Ages 76+ years**	10.4			9.2	
**SNHL progression, pure-tone audiometry (PTA) increase in dB, mean (SD)**					
** Ages 35-65 years**	1.1 (0.4)				^19^
** Ages 65+ years, PTA <40 dB HL**	1.4 (0.4)				
**Annual probability of hearing aid (HA) uptake (%)^a^**					
	PTA < 40 dB HL		PTA ≥ 40 dB HL		
** Ages 40-55 years**	0.5		2.3		^20^ ^,^ ^21^
** Ages 56-65 years**	0.5		4.6		
** Ages 66-75 years**	0.6		8.1		
** Ages 76-85 years**	0.7		7.2		
**Probability of HA discontinuation (%), yearly^a^**					
** 1 year after use**	12.9				^23^ ^,^ ^24^
** 10+ years after use**	3.5				
**Probability of cochlear implant (CI) implantation (%), yearly**					
** Adults with severe+ HL with HAs**	1.3				^22^
**Age-associated dementia clinical input parameters**					
**Annual incidence of dementia (%)^b^**					
	Male			Female	
** Ages 60-64 years**	0.4			0.3	^1^ ^,^ ^3^ ^,^ ^15^
** Ages 65-69 years**	0.7			0.4	
** Ages 70-74 years**	1.0			0.7	
** Ages 75-79 years**	1.7			1.6	
** Ages 80-84 years**	3.5			3.6	
** Ages 85-89 years**	4.2			6.0	
** Ages 90+ years**	5.9			7.1	
**Progression of dementia, mean years (SD)**					
** Mild to moderate dementia**	3.7 (3.1)				^3^ ^,^ ^25^ ^,^ ^26^
** Moderate to severe dementia**	2.0 (1.4)				
**Increased risk of death among people with dementia, HR**					
** Mild dementia**	2.2				^3^ ^,^ ^27^
** Moderate dementia**	3.1				
** Severe dementia**	5.0				
**Risk of developing dementia when HL ≥ 40 dB HL**					
** Incidence risk ratio**	2.0				Derived from^1^ ^,^ ^5^ ^,^ ^13^ ^,^ ^15^
**Health state utility input parameters**					
**Age-stratified base utilities^c^**					
** Base utility at age 40 years**	0.88				Derived from^8^ ^,^ ^28^
** Utility function based on age**	−2 × 10^−6^(age)^2^ − 0.0024(age) + 0.9834				
**Hearing loss utilities**					
**Utility decrements^d^**					
** Mild HL**	0.13				^29^ ^,^ ^30^
** Mild-moderate HL**	0.16				
** Moderate HL**	0.19				
** Moderate-severe HL**	0.26				
** Severe HL**	0.30				
** Profound HL**	0.31				
**Utility benefits**					
** Traditional hearing aids**	+0.11				^30^
** Cochlear implants**	+0.16				^30^
**Age-associated dementia utilities**					
**Utility decrements**					
** Mild dementia**	0.04				^8^ ^,^ ^28^
** Moderate dementia**	0.19				
** Severe dementia**	0.42				
**Cost input parameters**					
**Hearing loss costs, 2022 USD**					
** Audiology diagnostic test**	300				^31^
** Traditional hearing aid device(s)**	3690				^32^ ^,^ ^33^
** Annual traditional hearing aid costs after year 1**	910				^32^ ^,^ ^33^
** Cochlear implantation**	54 380				^31^ ^,^ ^34^
** Annual cochlear implant costs after year 1**	1260-1400				
**Age-associated dementia costs, 2022 USD**					
** Annual mild dementia**	12 590				^35^ ^,^ ^36^
** Annual moderate dementia**	15 290				
** Annual severe dementia**	45 170				

Abbreviations: CI, cochlear implant; dB HL, decibel in hearing level; HA, hearing aid; HL, hearing loss; dementia, age-associated dementia; HR, hazard ratio; PTA, pure tone average; SNHL, sensorineural hearing loss.

a In-between values in model are linearly interpolated.

b Values have been adjusted to remove dementia probability due to HL.

c Values have been adjusted to remove utility decrements due to dementia. Additionally, these values do not include decrements due to HL.

d HL levels for health state utilities are as follows: – Mild HL is defined in the model as hearing loss >25 dB and ≤34 dB HL. – Mild-moderate HL is defined in the model as hearing loss >34 dB and ≤49 dB HL. – Moderate HL is defined in the model as hearing loss >49 dB and ≤64 dB HL. – Moderate-severe HL is defined in the model as hearing loss >64 dB and ≤79 dB HL. – Severe HL is defined in the model as hearing loss >79 dB and ≤94 dB HL. – Profound HL is defined in the model as hearing loss >94 dB HL.

#### Natural history of dementia

We incorporated the yearly probability of incident, age-/sex-stratified dementia from the national Adult Changes in Thought cohort (range 0.3%-7.1% per year).^1^^,^^3^^,^^15^ We selected this cohort because it screened community-dwelling older adults for incident dementia using the Cognitive Abilities Screening Instrument, followed by a complete neuropsychological evaluation to confirm or refute a diagnosis of dementia for people who screen positive for dementia. We derived hearing loss-specific dementia incidence by adjusting a literature-reported prevalence risk ratio and attaining an dementia incidence risk ratio (IRR = 2.0) and incorporating the prevalence of moderate or greater hearing loss (defined as PTA thresholds ≥40dB HL; see Supplementary 2 for details on derivation of the incidence risk ratio).^1^^,^^5^ For dementia risk, we did not distinguish between sensorineural, conductive, or mixed hearing loss to remain consistent with the source estimates of dementia risk given hearing loss. Simulated individuals with dementia are then assigned a mean time period for the mild to moderate dementia transition (3.7-year average, SD = 3.1 years) and for moderate to severe dementia (2.0-year average, SD = 1.4 years).^3^^,^^25^^,^^26^ Simulated persons with dementia have an increased risk of mortality that increases with dementia severity.^3^^,^^27^ Mortality multipliers in the model were 2.2, 3.1, and 5.0, for simulated individuals with mild, moderate, and severe dementia and were applied to dementia-deleted “background” mortality.^27^

#### Non-dementia mortality

In the model, simulated persons experience annual probabilities of death from any cause other than dementia, using US lifetables (Supplementary 3).^38^ In concordance with recent evidence, and to remain conservative in our estimates of the lifetime quality of life effects of hearing loss and dementia, we assumed that hearing loss does not affect mortality directly.^39^^,^^40^ Inclusion of increased mortality associated with hearing loss would increase the effect of hearing loss and associated dementia on lifetime quality of life by shortening life expectancy. However, if hearing loss portends a competing risk of mortality alongside dementia, this assumption might lead us to overestimate the absolute number of dementia cases attributable to hearing loss. To address this, we include a sensitivity analysis wherein hearing loss directly impacts mortality.

#### Health state utilities

The disutility of hearing loss, stratified by severity, ranged from 0.13 to 0.31.^29^^,^^30^ The utility benefit of traditional (prescription) hearing aids and cochlear implants are +0.11 and +0.16.^30^^,^^41–43^ dementia-related health states utilities are also based on disease severity, with utility decrements of 0.04, 0.19, and 0.42, for mild, moderate, and severe dementia.^8^^,^^28^ In the base case, we assumed additive disutilities for hearing loss and dementia and varied this assumption in sensitivity analyses. Hearing loss and dementia disutilities were subtracted from a baseline utility based on age [eqn (1) where *y* is dementia-deleted quality of life, and *x* is age].^8^^,^^28^


(1) $$y=(-2\times 10^{-6})x^{2}-0.0024x+0.9834$$

#### Healthcare costs

We incorporated hearing and dementia-associated healthcare costs within the model. Hearing-associated costs included the audiology diagnostic test cost for prescription hearing aid acquisition only ($300), costs of hearing aids ($3690 one-time, $910 yearly recurring), and costs of cochlear implantation ($54 380 one-time, $1260-1400 yearly recurring).^14^^,^^32–34^ Dementia costs, including outpatient care, inpatient care, home care, medications, and long-term care were incorporated yearly and stratified by disease severity ($12 590 for mild dementia, $15 290 for moderate dementia, and $45 170 for severe dementia).^35^^,^^36^

### Model validation

After deriving age-/sex-stratified dementia incidence rates from the Adult Changes in Thought cohort, we validated model-projected annual dementia incidence against age-stratified annual dementia incidence from the Framingham Heart Study.^15^^,^^44^ We considered a root mean squared error (RMSE) of ≤15% to be acceptable.

### Sensitivity analysis

We varied key parameters across plausible ranges in deterministic sensitivity analyses. When possible, we varied individual parameters across their reported 95% confidence intervals. We also assigned distributions to select model parameters and performed a probabilistic sensitivity analysis on the percent of annual dementia cases attributable to hearing loss (see Supplementary 4 for input parameter distributions). We then performed 30 000 total model iterations, each iteration drawing from the input parameter distributions independently; we then collected the projected percent of annual dementia cases attributable to hearing loss.

## Results

### Dementia incidence

The expanded DeciBHAL model projected that in 2022 there would be 412 000 incident dementia cases in males (annual incidence of 1.2% given 34.1 million males at risk for dementia in the United States) and 523 000 incident dementia cases in females (annual incidence of 1.3% given 40.1 million females at risk). Annual dementia incidence would increase at each age stratum, ranging from 0.4%-5.4% (ages 60-64 to ages 85+) in males and 0.3%-7.3% (ages 60-64 to ages 85+) for females. Comparing DeciBHAL-projected dementia incidence with the Adult Changes in Thought incidences, the RMSE was 7.61. We then compared DeciBHAL-projected dementia incidence to the Framingham Heart Study literature reported age-stratified incidence of dementia.^44^ We found the DeciBHAL-projected dementia incidence to be within the 95% confidence intervals of the Framingham Heart Study dementia incidence at each age bucket (65-69, 70-74, 75-79, 80-84, 85-89 years).

### Hearing loss-attributable dementia

The model-projected dementia cases attributable to hearing loss in 2022 would be 73 000 for males (17.7% of all male dementia cases) and 68 000 for females (13.0% of all female dementia cases). Table 2 shows the annual incident dementia cases projected for the simulation that included hearing loss (HL+) and the counterfactual simulation without any hearing loss (HL−), where the difference between the two is the number of hearing loss-attributable dementia cases. In general, the proportion of hearing loss-attributable dementia cases increases with advancing age given rising hearing loss prevalence. Model projections showed that males have a higher attributable proportion of dementia due to higher hearing loss prevalence among males than females.

**Table 2.** Model-projected numbers of incident cases of dementia in the United States attributable to hearing loss by age and sex in 2022.

	Male			Female		
Age (years)	At-risk population^45^	Incident dementia cases (% of at-risk population)	Dementia cases attributable to HL (% of all male dementia cases)	At-risk population^45^	Incident dementia cases (% of at-risk population)	Dementia cases attributable to HL (% of all female dementia cases)
**60-64**	10 401 000	44 000 (0.4)	2000 (4.5)	11 015 000	35 000 (0.3)	1000 (2.9)
**65-69**	8 496 000	58 000 (0.7)	5000 (8.6)	9 541 000	34 000 (0.4)	1000 (2.9)
**70-74**	6 684 000	66 000 (1.0)	10 000 (15.2)	7 825 000	57 000 (0.7)	4000 (7.0)
**75-79**	4 409 000	73 000 (1.7)	15 000 (20.5)	5 606 000	86 000 (1.5)	10 000 (11.6)
**80-84**	2 379 000	77 000 (3.2)	19 000 (24.7)	3 271 000	105 000 (3.2)	17 000 (16.2)
**85+**	1 756 000	94 000 (5.4)	22 000 (23.4)	2 817 000	206 000 (7.3)	35 000 (17.0)
**Total**	34 125 000	412 000 (1.2)	73 000 (17.7)	40 075 000	523 000 (1.3)	68 000 (13.0)

Abbreviations: dementia, age-associated dementia; HL, hearing loss.

### Quality of life

Mean per-person undiscounted QALYs from age 40 years are 27.30 QALYs (males) and 31.02 QALYs (females). In the simulation without hearing loss, QALYs increase to 28.99 QALYs and 32.40 QALYs, indicating that preventing all hearing loss and hearing loss-associated dementia would increase the average per-person lifetime QALYs by 1.69 and 1.38 in males and females, respectively.

### Costs

The lifetime per-person average costs for hearing loss diagnosis and treatment and dementia, averaged amongst the entire cohort with and without hearing loss or dementia, would be $28 200 and $36 000 for males and females, respectively. In the HL- simulation, there are no hearing loss-related costs, and the lifetime per-person average costs for dementia alone (again averaged across persons with/without dementia) are reduced to $22 500 for the male cohort and $31 500 for the female cohort (20% and 13% reduction in costs, respectively).

### Deterministic sensitivity analyses

When hearing loss incidence is 120% of its basecase value, the number of attributable dementia cases in 2022 would increase to 82 000 in males (19.5% of all male dementia cases) and 75 000 in females (14.1% of all female dementia cases). When hearing loss incidence is 90% of its base case value, only 68 000 males (16.7% of all male dementia cases) and 60 000 females (11.7% of all female dementia cases) would be projected to develop dementia in 2022. When the incidence risk ratio of acquiring dementia due to hearing loss is varied (RR of 1.5-2.5), the yearly dementia cases in 2022 attributable to hearing loss range from 43 000-94 000 in males and 37 000-80 000 in females. Lastly, we included a sensitivity analysis in which hearing loss itself (outside of impacts through dementia) increases mortality by 13%/year.^40^ In this analysis, yearly dementia cases attributable to hearing loss in 2022 would decrease to 15.0% in males and 11.8% in females.

**Figure 2.**
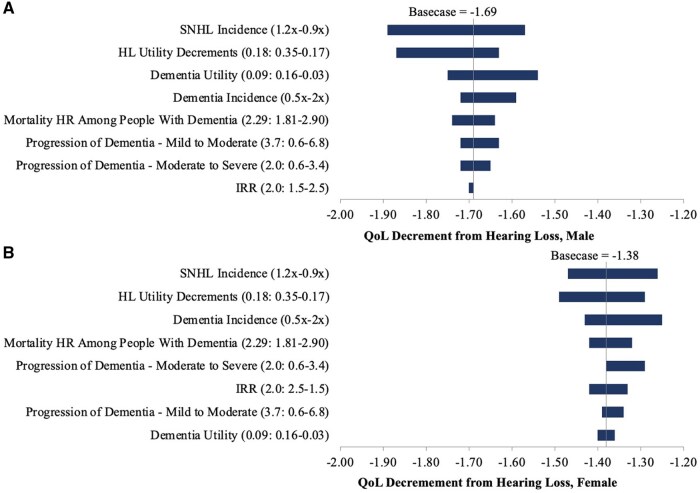
One-way sensitivity analyses for the lifetime quality of life decrements due to hearing loss and associated dementia. This tornado diagram shows the effects of varying single parameters across their plausible ranges on the lifetime quality-of-life decrement due to hearing loss and dementia. The length of each bar represents the difference in QoL decrements for the upper and lower values of each parameter varied in the sensitivity analysis. Panel A shows the sensitivity analysis results for males, where the basecase is −1.69, and Panel B shows the sensitivity analysis results for females, where the basecase is −1.38. HL, hearing loss; HR, hazard ratio; IRR, incidence risk ratio; QoL, quality of life; SNHL, sensorineural hearing loss.

We varied several parameters across their plausible ranges to determine the quality-of-life effects of hearing loss over a lifetime (Figure 2). Among these parameters, QoL decrements in males are most sensitive to variations in sensorineural hearing loss incidence, hearing loss utility decrements, and dementia utility. In females, QoL decrements are also most sensitive to variations in hearing loss incidence and hearing loss utility decrements, as well as variations in dementia incidence.

### Probabilistic sensitivity analysis

**Figure 3.**
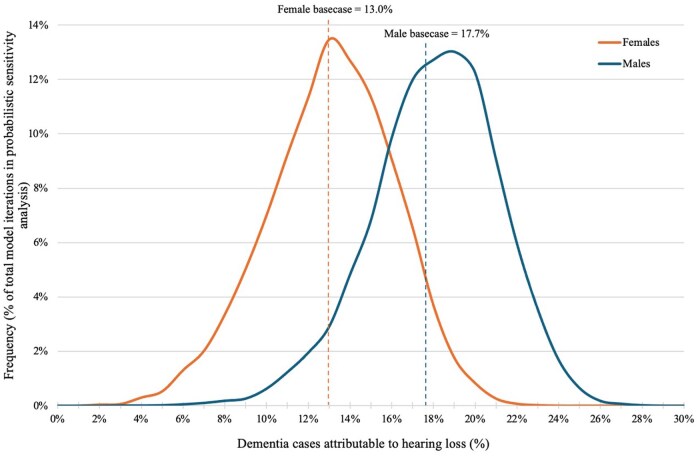
Sex-stratified distribution of dementia cases attributable to hearing loss in a probabilistic sensitivity analysis. This figure shows the results of 30 000 total model simulations where key input parameters were varied iteratively based on assigned distributions. The *x*-axis represents the percent of dementia cases attributable to hearing loss for each simulation, and the *y*-axis represents the frequency of the determined percent attributable in the PSA (i.e., percent of model iterations). The solid orange line (curve to the left) represents the distribution for the percent of female dementia cases attributable to HL, whereas the solid blue line (curve to the right) represents the distribution for males. The dashed orange (*x* = 13.0%) and blue lines (*x* = 17.7%) represent the basecase values for percent of dementia cases attributable to HL for females and males, respectively. HL, hearing loss; IRR, incidence risk ratio.

In probabilistic sensitivity analysis, 95% of the projected percentages of dementia cases attributable to hearing loss range between 11.5%-23.6% for males and 6.7%-18.7% for females (Figure 3).

## Discussion

This model-based analysis projected that 141 000 incident dementia cases in 2022 could have been attributable to hearing loss, which would account for 17.7% and 13.0% of incident dementia cases in 2022 amongst males and females in the United States. The difference in the proportion of dementia cases attributable to hearing loss between males and females in this analysis is attributable to the higher incidence and prevalence of hearing loss among males than females, which has both biologic and sociologic foundations.^46^^,^^47^ In terms of quality-of-life, hearing loss and associated dementia would reduce lifetime undiscounted QALYs by 1.69 and 1.38 in males and females in the United States, respectively. In sensitivity analyses, we found that these results are most sensitive to the risk ratio of dementia attributable to hearing loss. In probabilistic sensitivity analysis, 95% of model iterations placed the percent of dementia cases attributable to hearing loss within the range of 11.5%-23.6% for males and 6.7%-18.7% for females. In terms of the economic impact, we found that hearing loss and associated dementia would contribute on average $5700 and $4500 to lifetime healthcare costs for United States males and females.

These findings align with prior research that positions hearing loss among the leading modifiable factors contributing to dementia in the United States and globally.^4^ A recent US-based study using NHANES data to determine the population attributable fraction of dementia from hearing loss found that 17% of dementia cases may be attributable to hearing loss,^10^ and a study using Atherosclerosis Risk in Communities Neurocognitive Study (ARIC-NCS) similarly found ∼17% of hearing loss may be attributable to moderate or greater hearing loss.^9^ Globally, an influential report from the Lancet Commission on Dementia used meta-analysis and population attributable fraction methodologies to project that 7% of dementia worldwide is attributable to hearing loss.^4^ These studies used PAF methods that incorporate the prevalence of hearing loss in the population and the adjusted hazard ratio for incident dementia to determine the proportion of incident dementia cases attributable to hearing loss. Our decision modeling approach also takes into account the age- and sex-stratified incidence of hearing loss and dementia, as well as mortality associated with dementia. Despite the methodologic differences, our estimates of 17.7% for males and 13.0% for females of all yearly dementia cases attributable to hearing loss are similar to the US-based studies. Our estimates are higher than those estimated by the Lancet Commission, likely due to longer life expectancy in the United States compared to low- and middle-income countries (given that hearing loss incidence increases substantially with age), as well as methodologic differences whereby the Lancet Commission study corrected their PAF estimate for commonality between dementia risk factors.^4^^,^^9^^,^^48^ Additionally, the global relative risk for dementia in the Lancet analysis was 1.4, which was lower than the US-specific relative risk of 2.0 that we incorporated as a model input in this analysis.^1^^,^^4^^,^^5^^,^^13^^,^^15^ We varied this input parameter extensively in sensitivity analyses.

As hearing loss cannot be randomized, the highest level of ascertainable evidence on the association between hearing loss and dementia will be observational in nature. With mounting evidence associating hearing loss with incident dementia, the precise role of hearing loss as a causative factor for dementia has been called into question.^49^ Potential mechanisms whereby hearing loss could cause or worsen dementia directly include neurobiological changes in synaptic connections in the brain, as well as indirect social mechanisms such as increased social isolation.^50^ This theory is supported by numerous observational studies that control for other confounding factors that can increase dementia risk and demonstrate that the effects of hearing loss on the risk of dementia persist.^4^ Yet others posit that this association may be weakened, or negated, by hypotheses that hearing loss and dementia may be related and share a common cause rather than have a causal association.^49^ That said, simulation models can be useful to help examine the potential impact of associations that cannot be randomized.

Prevention of hearing loss across the lifespan includes limiting exposure to loud sounds, appropriate use of ototoxic medications, increased uptake of immunizations for infections that can cause hearing loss, and prompt identification and management of treatable ear diseases including otitis media.^51^ Our analysis projects the maximum yearly number of dementia cases that could be averted if all hearing loss were prevented. However, current hearing loss prevention measures are likely to prevent only a portion of hearing loss—and therefore, only reduce a proportional number of hearing loss-associated dementia cases. One potential prevention target is reducing loud sound exposure, with billions of persons exposed to loud sounds throughout their lifetime.^52^ While the exact effects of repeated loud sound exposures on hearing loss later in life remain to be elucidated, some research suggests that excessive exposure could accelerate age-related changes to hearing.^53^ Previous research has estimated that up to 7% of hearing loss might be attributable to noise exposure, indicating that up to 10 000 dementia cases could be prevented yearly by eliminating noise-induced hearing loss.^54^ Our results demonstrate that if hearing loss incidence is reduced by 10%, up to 13 000 dementia cases attributable to hearing loss could be prevented each year. Future research should focus on quantifying the increased risk of hearing loss associated with cumulative loud sound exposure throughout the lifespan, and the potential effects of interventions to reduce loud sound exposure on hearing loss risk.

Our study also showed that hearing loss and associated dementia have substantial effects on quality-adjusted life-expectancy. Averaged across all persons with and without hearing loss or dementia, quality-adjusted life-expectancy from age 40 years is reduced by 1.38 and 1.69 years for females and males, respectively, potentially representing years of independent living lost: Interventions that can prevent hearing loss and dementia or improve quality of life in those impacted will be critical. For the millions of Americans who have hearing loss, effective treatments exist including traditional (prescription) and over-the-counter hearing aids, cochlear implants, and aural rehabilitation that would improve quality of life and may prevent or slow neurobiological and psychosocial consequences that may predispose to dementia onset or progression.^4^^,^^30^^,^^49^^,^^55^ Identification of hearing loss, integetrated into routine primary care, geriatrics care, or dementia prevention programs, through routine screening schedules or symptomatic presentation to care, along with timely connection to hearing-related healthcare services will prove critical in linking people to effective treatments.^16^ Addressing hearing loss and cognitive impairment in older adults requires interdisciplinary collaboration among audiology, geriatrics, neurology, and primary care, to ensure coordinated care for this vulnerable population. Prescription hearing aids for people with all severities of hearing loss, and cochlear implants for people with moderate and profound losses, can improve hearing related outcomes and overall health-related quality of life.^14^^,^^30^^,^^56^ There is mixed evidence of the efficacy of hearing aids in preventing dementia onset or ­progression;^55^ however, there is substantial evidence around the efficacy of hearing aids to improve quality of life and social interactions.^30^^,^^56^ To the extent that there is any benefit of hearing aid use on preventing the onset or worsening of dementia, hearing aids represent a safe, increasingly utilized, and relatively low-cost intervention that concomitantly would improve health-related quality of life.^56^

Our study has several limitations. First, these model-based results are dependent on the adjusted relative risk of acquiring dementia attributable to hearing loss.^1^^,^^5^^,^^13^^,^^15^ This association has been replicated in several studies but does not necessarily represent a causal association. Indeed, risk factors for hearing loss have many co-occurring risk factors for dementia, including non-audiological factors that reflect the complexity of aging such as frailty, multi-morbidity, and social determinants of health, or changes, including those related to social mechanisms. Our model does not account for these factors which could have independent or mediating effects on the relationship between hearing loss and dementia and could result in elevated estimates of associations between hearing loss and dementia. However, to overcome this and other limitations, we incorporated the best available data and examined the uncertainty in sensitivity analysis that lowered the association between hearing loss and dementia, including probabilistic sensitivity analysis. Second, there is burgeoning evidence that hearing aids could prevent or slow cognitive decline in people most at risk for dementia.^55^ In the current analysis, we did not incorporate the effects of hearing aid use on cognition given the preliminary nature of the data. In future analyses, we plan to project the potential caseload of dementia that could be averted with increases in hearing aid use. Third, we did not project the cost-effectiveness of hearing loss or dementia interventions because it is out of scope of this analysis. In future research, we will explore the economic effects of hearing loss and associated dementia in more detail. Lastly, due to data limitations, we did not include differences in hearing loss or dementia by race, ethnicity, and socioeconomic factors that affect the social determinants of health. While persons of White race are known to have a higher prevalence of hearing loss compared to persons of other races in the United States, it is not clear how the risk of dementia attributable to hearing loss may vary among persons of different races and ethnicities.^57^ Additionally, access to hearing aids is lower among Black and Hispanic persons which may affect outcomes of hearing-related quality of life and dementia risk.^58^^,^^59^

In conclusion, we projected that 141 000 incident dementia cases occurring in the United States in 2022 would have been potentially attributable to hearing loss. These results highlight the public health importance of preventing hearing loss when possible, and the value of early identification and treatment of hearing loss to improve quality of life and avert dementia cases in the United States.

## Supplementary Material

glaf295_Supplementary_Data

## Data Availability

All data relevant to the study are included in the article. Original model code is available upon request. Interested readers should contact Dr. Ethan Borre (eborre@mgh.harvard.edu).
