# Supplementary material for: The impact of hearing loss on annual incident age-associated dementia cases and quality of life in the United States
Source: J Gerontol A Biol Sci Med Sci. 2026 Feb 3;81(5):glaf295. doi: 10.1093/gerona/glaf295 (PMC13064983; doi:10.1093/gerona/glaf295)
Supplement: glaf295_Supplementary_Data [file glaf295_supplementary_data.docx]

**The Impact of Hearing Loss on Annual Incident Age-Associated Dementia Cases and Quality Adjusted Life Expectancy in the US**

**Supplementary Appendix**

Ethan D. Borre, MD, PhD

Julie N. Deleger, BA

Lauren K. Dillard, AuD, PhD

Juliessa M. Pavon, MD

Sachin J. Shah, MD, MPH

Judy R. Dubno, PhD

Sherri L. Smith, PhD

Kenneth A. Freedberg, MD, MSc

Howard W. Francis, MD, MBA

Christine S. Ritchie, MD, MSPH

Gillian D. Sanders Schmidler, PhD

Emily P. Hyle, MD, MSc

**CONTENTS**

[**Supplement 1. Internal and external validation of the age-associated dementia module in DeciBHAL** 2](#_Toc185342468)

[**Supplement 2. Derivation of hearing-loss specific age-associated dementia incidence.** 3](#_Toc185342469)

[**Supplement 3. Derivation of age-associated dementia-specific background mortality** 4](#_Toc185342470)

[**Supplement 4. Model input distributions for probabilistic sensitivity analysis.** 5](#_Toc185342471)

[eTable 1. Probabilistic sensitivity analysis model input parameter ranges 5](#_Toc185342472)

[**References** 6](#_Toc185342473)

# **Supplement 1. Internal and external validation of the age-associated dementia module in DeciBHAL**

To incorporate age-associated dementia (AAD) in DeciBHAL, we integrated model structure and inputs from a previously validated model of aging and AAD (1,2). We then performed internal and external validation exercises on the expanded DeciBHAL model.

First, we reviewed model structure and inputs with subject matter experts in hearing loss, aging, and AAD (Figure 1). We then ensured that the hearing loss prevalence at ages 55, 65, 75, and 85 years matched the literature values used to derive the hearing loss incidence from the National Health and Nutrition Examination Survey (3).

Second, we ensured that dementia incidence projected by DeciBHAL matched the age- and sex-stratified incidence of dementia from the Adult Changes in Thought cohort (ACT) that was used to derive these inputs (4). Our validation exercise comparing DeciBHAL-projected AAD incidence with the ACT incidences had a root mean squared error (RMSE) of 7.63 (we considered RMSE≤15% acceptable). We then compared DeciBHAL-projected AAD incidence to the Framingham Heart Study literature-reported age-stratified incidence of dementia (5). We found that the DeciBHAL-projected AAD incidence was within the 95% confidence intervals of the Framingham Heart Study AAD incidence at each age bucket (65-69, 70-74, 75-79, 80-84, 85-89 years).

Lastly, we calibrated the incidence risk ratio (IRR) of AAD attributable to hearing loss (2–4,6); please refer to Supplement 2 for further details.

# **Supplement 2. Derivation of hearing loss-specific age-associated dementia incidence.**

We derived hearing loss-specific age-associated dementia incidence incorporating the incidence of dementia in all persons, the prevalence of hearing loss, and the incidence risk ratio for dementia attributable to hearing loss. We first used DeciBHAL to identify the prevalence of hearing loss (40 dB HL or greater) for males and females at ages 62, 67, 72, 77, 82, 87, and 95 years. These model-projected prevalences were previously validated using National Health and Nutrition Examination Survey (NHANES) data (3).

We used these age-specific hearing loss prevalences to determine the AAD incidence in persons with and without hearing loss. Population level AAD incidence was from the ACT Cohort (4). We incorporated an adjusted prevalence risk ratio (PRR) for current dementia that is 1.61x higher for people with HL than people without (6). We first assumed that the prevalence risk ratio = the incidence risk ratio, and modified this assumption in later analyses. For these calculations, we used the following formula, where “mspHL” indicates all people with hearing losses at least 40 dB HL or greater:

$\frac{population level dementia incidence}{\left( 1-mspHL \right)+\left( IRR*mspHL \right)}$ $=HL-deleted AAD incidence$

Once these values were calculated for every age and sex, they were imputed into the model such that persons without HL (or hearing loss 39 dB HL or better) received the HL-deleted AAD incidence, and persons with HL 40 dB HL or worse received the HL-specific AAD incidence.

Lastly, we calibrated the IRR in the model to the PRR from the literature of 1.61 at a mean age of 81.1 years, which was the mean age of the study cohort (6).

$$PRR in model=\frac{prevalence of AAD in people WITH mspHL}{prevalence of AAD in people WITHOUT msphHL}$$

$=\frac{\frac{\# alive with AAD \& mod+HL}{\# alive with mod+HL}}{\frac{\# alive with AAD \& NO mod+HL}{\# alive without mod+HL}}$

We repeated this process until the IRR resulted in a PRR of 1.61. These calibrations resulted in an IRR of 2.0.

# **Supplement 3. Derivation of age-associated dementia-specific background mortality**

In DeciBHAL, the yearly probability of death can be from one of two causes: AAD-associated mortality and non-AAD-associated mortality. To avoid double-counting AAD -associated death, we derived AAD-associated and non-AAD-associated life-tables. We used the Centers for Disease Control and Prevention’s National Center for Health Statistics National Vital Statistics System’s cause-of-death data and the Human Mortality Database to define the US population size (7,8). To derive these life-tables, we defined all-cause death as if any cause of death was recorded and AAD-associated death if any dementia-related cause of death was recorded. We then used a previously fitted SAS program to generate AAD-associated and non-AAD-associated lifetables (9). This derivation method has been performed in a previously-validated model on aging and age-associated dementia (1).

# **Supplement 4. Model input distributions for probabilistic sensitivity analysis.**

## **eTable 1. Probabilistic sensitivity analysis model input parameter ranges**

| **Input Parameter** | **Distribution** | **Value (based on 95% confidence intervals, adjusted if needed)** | | **Reference** |
| --- | --- | --- | --- | --- |
|  |  | **Lower-bound** | **Upper-bound** |  |
| **Incidence risk ratio** | Normal | 1.5 | 2.5 | (2–4,6) |
| **SNHL incidence** |  |  |  |  |
| Male |  |  |  |  |
| 40-45 | Beta | 0.0049 | 0.0104 | (3,10–12) |
| 46-55 |  | 0.0089 | 0.0154 |  |
| 56-65 |  | 0.0194 | 0.0273 |  |
| 66-75 |  | 0.0486 | 0.0591 |  |
| 75+ |  | 0.0999 | 0.1085 |  |
| Female |  |  |  |  |
| 40-45 | Beta | 0.0004 | 0.0008 | (3,10–12) |
| 46-55 |  | 0.0026 | 0.0046 |  |
| 56-65 |  | 0.0104 | 0.0146 |  |
| 66-75 |  | 0.0345 | 0.0420 |  |
| 75+ |  | 0.0879 | 0.0955 |  |
| **AAD incidence** |  |  |  |  |
| Male |  |  |  |  |
| 60-64 | Beta | 0.0018 | 0.0103 | (1,3,4) |
| 65-69 |  | 0.0028 | 0.0162 |  |
| 70-74 |  | 0.0067 | 0.0142 |  |
| 75-79 |  | 0.0129 | 0.0213 |  |
| 80-84 |  | 0.0294 | 0.0426 |  |
| 85-89 |  | 0.0332 | 0.0532 |  |
| 90+ |  | 0.0421 | 0.0825 |  |
| Female |  |  |  |  |
| 60-64 | Beta | 0.0010 | 0.0313 | (1,3,4) |
| 65-69 |  | 0.0012 | 0.0363 |  |
| 70-74 |  | 0.0050 | 0.0165 |  |
| 75-79 |  | 0.0128 | 0.0254 |  |
| 80-84 |  | 0.0311 | 0.0497 |  |
| 85-89 |  | 0.0510 | 0.0819 |  |
| 90+ |  | 0.0575 | 0.1068 |  |

Abbreviations: SNHL, sensorineural hearing loss; AAD, age-associated incidence

# **References**

1. Hyle EP, Foote JHA, Shebl FM, Qian Y, Reddy KP, Mukerji SS, et al. Development and validation of the age-associated dementia policy (AgeD-Pol) computer simulation model in the USA and Europe. BMJ Open. 2022 Jul;12(7):e056546.

2. Borre ED, Myers ER, Dubno JR, O’Donoghue GM, Diab MM, Emmett SD, et al. Development and validation of DeciBHAL-US: A novel microsimulation model of hearing loss across the lifespan in the United States. eClinicalMedicine. 2022 Feb;44:101268.

3. Goman AM, Lin FR. Prevalence of hearing loss by severity in the United States. Am J Public Health. 2016 Oct;106(10):1820–2.

4. Tom SE, Hubbard RA, Crane PK, Haneuse SJ, Bowen J, McCormick WC, et al. Characterization of dementia and Alzheimer’s disease in an older population: updated incidence and life expectancy with and without dementia. Am J Public Health. 2015 Feb;105(2):408–13.

5. Wolters FJ, Chibnik LB, Waziry R, Anderson R, Berr C, Beiser A, et al. Twenty-seven-year time trends in dementia incidence in Europe and the United States: The Alzheimer Cohorts Consortium. Neurology. 2020 Aug 4;95(5):e519–31.

6. Huang AR, Jiang K, Lin FR, Deal JA, Reed NS. Hearing loss and dementia prevalence in older adults in the us. JAMA. 2023 Jan 10;329(2):171.

7. CDC. Mortality Multiple Cause-of-Death [Internet]. 2019 [cited 2024 Aug 13]. Available from: https://www.cdc.gov/nchs/nvss/mortality_public_use_data.htm

8. Human Mortality Database - USA [Internet]. [cited 2024 Dec 17]. Available from: https://www.mortality.org/Country/Country?cntr=USA

9. Vincent A, Zheng S. “Life expectancy tables” getting SAS® to run the hard math.

10. Homans NC, Metselaar RM, Dingemanse JG, van der Schroeff MP, Brocaar MP, Wieringa MH, et al. Prevalence of age-related hearing loss, including sex differences, in older adults in a large cohort study. Laryngoscope. 2017 Mar;127(3):725–30.

11. Cruickshanks KJ, Wiley TL, Tweed TS, Klein BEK, Klein R, Mares-Perlman JA, et al. Prevalence of hearing loss in older adults in Beaver Dam, Wisconsin: the Epidemiology of Hearing Loss Study. Am J Epidemiol. 1998 Nov 1;148(9):879–86.

12. Van Naarden K, Decouflé P, Caldwell K. Prevalence and characteristics of children with serious hearing impairment in metropolitan Atlanta, 1991–1993. Pediatrics. 1999 Mar 1;103(3):570–5.
